# Chronic hypoparathyroidism and treatment with teriparatide

**DOI:** 10.1007/s12020-020-02577-x

**Published:** 2021-02-04

**Authors:** Gemma Marcucci, Laura Masi, Luisella Cianferotti, Francesca Giusti, Caterina Fossi, Simone Parri, Giorgio Gronchi, Maria Luisa Brandi

**Affiliations:** 1 Bone Metabolic Diseases Unit, Department of Experimental and Clinical Biomedical Sciences, University of Florence, University Hospital of Florence, Florence, Italy; 2 Bone Metabolic Diseases Unit, University Hospital of Florence, Florence, Italy; 3 Department of Neurosciences, Psychology, Drug Research, and Child Health, University of Florence, Florence, Italy

**Keywords:** Parathyroid hormone, hypocalcemia, rare disease, orphan drug, therapy, thyroidectomy

## Abstract

**Purpose:**

Chronic hypoparathyroidism is usually treated with calcium and active vitamin D metabolites or analogs, despite the fact that their chronic use can lead to long-term complications. The use of hormone replacement therapy with PTH peptides [teriparatide and rhPTH (1–84)] has therefore been proposed. The main purpose of this study was to investigate the efficacy of teriparatide dose at 20 µg once or twice daily, in order to maintain normocalcemia reducing standard treatment, in adult patients with chronic hypoparathyroidism not well controlled with conventional treatment.

**Methods:**

The study was a Phase III, open-label, non-comparative, clinical investigation (study period: 3 months), at a tertiary care clinical research center. Thirty patients with chronic hypoparathyroidism were screened, and 12 started teriparatide. After the optimization phase (0–4 weeks), calcium and calcitriol supplements were progressively reduced, while teriparatide 20 µg once daily was administered (5–7 weeks), and then could be titrated up to 20 µg twice daily (7–17 weeks). The main outcome measures included serum and urinary biochemical exams and Rand 36-Item Short Form Health Survey.

**Results:**

This study showed that teriparatide 20 µg once daily was insufficient to discontinue calcium and calcitriol supplements to maintain normal serum calcium concentrations. Conversely, for more than half of patients treated with teriparatide 20 µg twice daily, calcium and calcitriol administration was avoidable, but in some cases at the expense of serum calcium and phosphate oscillations.

**Conclusions:**

Since intervention trials evaluating the efficacy and safety of teriparatide in hypoparathyroid patients are not yet available, the routine use of this molecule poses some doubts.

## Introduction

Conventional treatment of chronic hypoparathyroidism (HypoPT) consists of calcium supplements and active vitamin D metabolites or analogs, whose chronic use at high doses can lead to long-term complications [1–4]. In the past two decades, synthetic human PTH (1–34) and recombinant human parathyroid hormone [rhPTH (1–84)], have been evaluated for understanding the potential role of these therapies in HypoPT.

In 2002, synthetic human PTH (1–34) was evaluated for the treatment of postmenopausal osteoporosis [5–7]. Subsequent studies confirmed the efficacy and safety of teriparatide in large groups of patients suffering from osteoporosis, also compared with other anti-fracture treatments [8, 9]. Regarding hypoparathyroidism, some small-sized trials have investigated its effects in hypoparathyroid patients not adequately controlled with conventional treatment [10–14]. The studies, which employed synthetic human PTH (1–34) in small samples of adult and pediatric patients with various HypoPT forms, included randomized open-label and crossover trials [10, 11, 14, 15]. In these studies, it was demonstrated that twice-daily injections of synthetic human PTH (1–34) (0.5 µg/kg·dose) allowed good control of serum calcium levels as compared to conventional therapy, without exhibiting a significant reduction in 24-h urinary calcium excretion [10, 14]. In studies conducted on 8 adult patients affected by postsurgical HypoPT and in 12 children and young adult patients with congenital HypoPT due to Autoimmune polyendocrine syndrome type 1 or *CaSR* gene mutations, the use of an infusion pump (mean dose in adult patients: 13 ± 4 µg/day) appeared to normalize serum calcium, phosphate, and bone biomarker concentrations, while significantly reducing 24-h urinary calcium excretion [11, 15].

Since the approval by the Food and Drug Administration (FDA) of human recombinant PTH (1–34) [rhPTH (1–34), teriparatide, Forteo^®^; Eli Lilly] for patients with severe postmenopausal osteoporosis, the occurrence of osteosarcomas and death in rat toxicology studies of rhPTH (1–34), brought about a limit in dosage (not > 20 µg/daily) and time of administration (not > 24 months) [16, 17]. Moreover, a warning was posed against the use of rhPTH (1–34) in pediatric patients or young adults with open epiphyses [16, 18, 19].

In 2013, the reimbursement of teriparatide therapy (from 20 to 80 µg/daily) was approved in Italy for patients affected by severe chronic HypoPT not adequately controlled with conventional treatment, with a limit then extended to three years. Subsequently, a spontaneous collection of data allowed the publication of prospective open-label investigations on 42 adult patients affected by postsurgical HypoPT treated with teriparatide (20 µg twice/daily) for a maximum period of 24 months [20, 21]. Until now, no clinical trials for registration on teriparatide treatment have been conducted in hypoparathyroid patients. Therefore, no efficacy and safety data for teriparatide are available in these patients, especially considering the prolonged use of doses greater than 20 µg daily, use in young subjects, even if over 18 years of age, and the potential risk of osteosarcoma.

On the other hand, rhPTH (1–84) represents the full-length hormone missing in chronic HypoPT. In Europe, at first rhPTH (1–84) was approved for the treatment of severe osteoporosis (100 µg/once daily s.c.) in adult patients, with a limit of 24 months of treatment [22]. Then, rhPTH (1–84) was withdrawn from the osteoporosis market for its lack of superiority *vs* teriparatide and the higher frequency of hypercalcemia and hypercalciuria [23, 24]. Since rhPTH (1–84) has a slightly longer half-life and a higher increase in calcemia than rhPTH (1–34), this could be advantageous in treating HypoPT [25]. In 2013, the results of the randomized, double-blind, placebo-controlled phase 3 clinical trial (“REPLACE”) on the use of rhPTH (1–84) in hypoparathyroid patients were published [24]. Subsequently, further studies assessed its efficacy, safety, and effects on quality of life in patients affected by chronic HypoPT [26–31]. Therefore, in 2015 the FDA approved the use of Natpara^®^ [rhPTH (1–84)] in the USA in patients with chronic HypoPT not adequately controlled by standard therapy, with a “black box” warning related to the potential risk of osteosarcoma, but without the time limit of use [32–36]. Two years later, the European Medicines Agency (EMA) recommended granting a conditional marketing authorization in the European Union (EU) for Natpar^®^ [rhPTH (1–84)].

The objective of this study was to investigate the efficacy and safety of teriparatide dose (20 µg once or twice daily), necessary to maintain normal calcium levels reducing (at least 50%) or suspending supplementation with calcitriol and calcium. This objective was evaluated in a period of 3 months in a sample of adult patients with chronic HypoPT not well controlled with conventional therapy. Unlike the other two studies performed with teriparatide [20, 21], using a device that does not allow a pro-kg dosage, this is the first study that shows results also with 20 µg once daily, currently recommended dosage, according to safety studies on osteoporotic patients [5–7, 22].

## Subjects and methods

### Study design

The study was a Phase III, open-label, non-comparative, single-center, clinical trial, conducted on adult subjects (aged ≥ 18 years) of both sexes with chronic HypoPT, treated with teriparatide subcutaneous (s.c.) injections 20 µg once or twice daily (Forsteo®). The study was divided into three phases: recruitment, optimization (4 weeks; visits 1–2), and treatment (13 weeks; visits 3–10). The study was carried out from June 2013 to June 2016 at the Metabolic Bone Diseases Unit of the University of Florence. It was part of a project “Rare Diseases 2008” of the Italian Ministry of Health, approved by the Investigator Board of the University Hospital of Florence and AIFA (Code-EUDRACT:2013-001890-26). All subjects participating in the study signed an informed consent form, and all personal, clinical, and biological data were collected in accordance with the Declaration of Helsinki.

The primary objective of the study was the evaluation of the required dose of teriparatide necessary to maintain normal calcium levels reducing (at least 50%) or suspending supplementation with calcitriol and/or calcium in patients with chronic HypoPT, not adequately controlled with standard treatment. The primary endpoints were: (1) to determine the proportion of patients that demonstrated at least a 50% reduction or total withdrawal of oral calcium supplement and active vitamin D analogs by the end of the teriparatide treatment period; (2) the proportion of patients in whom the treatment maintained stable serum calcium and 24-h urinary calcium within the normal range. The secondary endpoints included: (1) to evaluate the frequency of hypocalcemia episodes during the teriparatide treatment period and/or eventual adverse events; (2) to determine the change in score for quality of life as measured using the Short Form-36 (SF-36) questionnaire.

In the optimization phase, adequate supplementation of oral calcium, cholecalciferol or calcifediol, and calcitriol, was administered to maintain serum calcium levels within the normal range and optimal vitamin D status.

During the first 2 weeks of treatment with teriparatide, recruited patients were treated with teriparatide 20 µg once daily, reducing, as far as possible, the supplementation of oral calcium and calcitriol. On the first day of treatment, patients took teriparatide in the morning and concomitantly reduced their total daily dose of calcitriol and, if possible, also of calcium, by 50%. During the following days (days 2 or 3), a measurement of serum calcium was performed, and after 1 week, if possible, the supplementations with calcium and calcitriol were further reduced/eliminated. At the end of 2 weeks, the dosage of teriparatide could be titrated from 20 µg once daily up to twice daily, and concomitantly the total daily dose of calcitriol could be eliminated, and if possible, also the daily dose of oral calcium supplement. During the following days (days 16 or 17), another measurement of serum calcium was performed. Any adverse event was reported.

### Study population

The study evaluated 30 male and female adult patients (18–65 years; patients <25 years old were examined radiologically to ensure epiphyseal closure) with a history of chronic HypoPT, not adequately treated with conventional therapy according to inclusion criteria, which included: (1) history of chronic HypoPT lasting more than 18 months, inclusive of biochemical evidence of hypocalcemia and concomitant serum intact PTH < 1.3 pmol/L; (2) requirement for oral calcium supplements ≥1000 mg/day over normal dietary calcium intake; (3) no adequate compliance therapy or presence of long-term complications with conventional therapy; (4) capability of providing written informed consent; and (5) ability to perform daily subcutaneous self-injections of study drug (or have a designee perform injection) via an injection pen into the thigh or abdomen.

Exclusion criteria: (1) functional HypoPT resulting from impaired responsiveness to PTH; (2) any disease that might affect calcium metabolism or calcium/phosphate homeostasis other than HypoPT; (3) use of prohibited medications such as loop diuretics, raloxifene, hydrochloride, lithium, estrogens, progestins, methotrexate, or systemic corticosteroids within clinical trial optimization and treatment periods; (4) epilepsy; (5) seizure disorder with a history of a seizure within the previous 6 months before the study; (6) the presence of open epiphyses; and (7) any disease/condition that, in the opinion of the Investigator, had a high probability of precluding the patient from correctly following study requirements and/or completing the study.

### Assays, evaluation of BMD, and quality of life

Serum calcium, phosphate, albumin, creatinine and urinary calcium values, and creatinuria were determined by colorimetric method, and the phosphaturia by a potentiometric method. In the study, serum calcium values were corrected for albumin values using the following formula: serum calcium (mg/dL) + 0.8 × (4-serum albumin g/dL). Creatinine clearance was calculated from values of serum creatinine, and urine output creatinuria in the 24-h collection. Plasma PTH was determined by electro-chemiluminescence (ECLIA), while 25(OH)D and 1,25-dihydroxyvitamin D [1,25(OH)_2_D] by immunochemiluminescent methods. For the determination of bone alkaline phosphatase and urinary deoxypyridinoline, immunoassay and immunochemiluminescence were used, respectively. All blood and urine samples were measured at the University Hospital central laboratory. Bone mineral density (BMD) was assessed by DEXA (Dual-energy X-ray absorptiometry), at the lumbar spine (L-BMD) and hip (F-BMD) (Hologic, Discovery A, SN84699, version 13.3.3). The evaluation of the quality of life was performed by the SF-36 questionnaire.

### Statistical methods

Data are presented as mean ± SD (standard deviation), unless otherwise stated. Repeated measures-related differences were evaluated by using Student’s t-test for paired-sample (SPSS software). *P*-value of ≤ 0.05 was considered statistically significant (SPSS software).

On the basis of previous literature [10–15, 20, 21], a large effect size (Cohen’s *d* = 0.90) was assumed. With alpha = 0.05, a sample of at least 10 participants enables a power of 0.80.

## Results

### Study population

Thirty patients with chronic HypoPT were screened, 19 patients were eligible, according to inclusion and exclusion criteria, with the remaining 11 patients considered as screening failure. Of the 19 eligible and enrolled patients, 5 withdrew informed consent after the optimization phase, and 2 patients were excluded, before the treatment period with teriparatide, for elapsing suspected malignancy. Therefore, 12 patients were commenced on teriparatide, with 10 completing the period of treatment, while 2 patients stopped for adverse events.

### Baseline characteristics

The adult patient sample recruited for teriparatide treatment consisted of 7 females (58.3%) and 5 males. Nine patients had postsurgical HypoPT (75%), and 3 patients had idiopathic HypoPT (25%). Among patients with postsurgical HypoPT, 5 underwent thyroidectomy for thyroid goiter, 3 for Graves’ disease, and 1 for thyroid follicular carcinoma. The average duration of chronic HypoPT was 12.6 years (range: 2–36).

Recruited patients were not well controlled with conventional treatment. At baseline visit, patients reported after the diagnosis of HypoPT: frequent episodes of hypocalcemia, tingling/cramps/paresthesia despite conventional therapy (*n*:9); kidney stones (*n*:1), nephrocalcinosis (*n*:1), poor compliance to conventional therapy (*n*:3), basal ganglia calcifications (*n*:1), low concentration ability (*n*:1), and anxiety and panic attacks (*n*:1).

Table 1 summarizes general characteristics at baseline and the results of serum and urinary exams after the optimization phase (visit 3) and at the end of the teriparatide treatment period (visit 10), the biochemical data of 2 patients who discontinued on visits 6 and 7, as above specified, are not included.

**Table. 1:** General characteristics at baseline and serum and urinary exams performed at the beginning (visit 3) and at the end (visit 10) of the teriparatide treatment period

Clinical features (baseline)								
		Mean			SD		Range	
Age, years		48.6			18.4		24–73	
Body mass index		27.1			4.49		21–34.5	

DEXA (baseline)								
		BMD (g/cm^2^)			*T*-score		*Z*-score	
Total lumbar: mean ± SD		1.068 ± 0.18			0.08 ± 1.68		0.95 ± 1.6	
Total femoral: mean ± SD		0.90 ± 0.19			−0.2 ± 1.05		0.46 ± 1.0	
Femoral neck: mean ± SD		0.92 ± 0.20			−0.6 ± 1.09		0.19 ± 1.0	

Calcium carbonate and calcitriol doses (after optimization phase, visit 3)								
		Mean			SD		Range	
Calcium carbonate gr/day		1450			395		1000–2000	
Calcitriol µg/day		1.00			0.26		0.50–1.25	

Blood and urine exams after the optimization phase (visit 3) and at the end of the teriparatide treatment period (visit 10)								
Exam	Reference range/unit of measures		Mean ± SD visit 3	Mean ± SD visit 10		Paired-sample Student’s *t*		*P*-value (*<0.05)
s Ca corrected for albumin	8.5–10.1 mg/dL		7.68 ± 0.80	8.57 ± 0.94		−3.34		0.010*
s P	2.5–4.9 mg/dL		4.43 ± 0.54	4.36 ± 0.79		0.85		0.421
24-h u Ca	42–353 mg/24 h		278.82 ± 143.90	290.66 ± 120.90		0.41		0.695
24-h u P	400–1300 mg/24 h		640.50 ± 305.16	999.07 ± 706.27		−0.33		0.753
1,25(OH)_2_D_3_	26–95 pg/mL		41.85 ± 17.12	69.87 ± 37.16		−3.03		0.023*
25(OH)D	30–100 ng/mL		27.85 ± 5.42	18.3 ± 6.20		3.11		0.017*
Creatinine clearance	>60 mg/dL		79.39 ± 11.82	81.94 ± 8.17		−2.77		0.033*
BALP	men: 7–20.1 µg/L, pre-menopause women: 4–14.3 µg/l; post-menopause women: 6–22.5 µg/L		11.56 ± 3.82	33.20 ± 13.44		−4.77		0.003*
u DPD	3–7.4 nmol/mmol		4.80 ± 1.36	10.6 ± 3.90		−3.39		0.015*
Ca-P product	35–55 mg^2^/dL^2^		32.02 ± 8.1	36.36 ± 6.29		−2.07		0.069

Paired-sample Student’s *t*: a negative value of *t* indicates an increase in values between visit 3 and visit 10, while the positive value of *t* indicates a decrease in values between visit 3 and visit 10. *P-value < 0.05

In this table, the biochemical data of two patients who discontinued on visit 6 and 7 were not inserted

*s* serum, *u* urinary, *Ca* calcium, *P* phosphate, *BALP* bone alkaline phosphatase, *DPD* deoxypyridinoline

### Teriparatide and oral calcitriol and calcium supplement doses

All 12 patients at the beginning of teriparatide treatment started teriparatide 20 µg once daily. This dosage was not sufficient to reduce at least 50% or withdraw calcium and calcitriol supplementation; the dose was therefore increased to 20 µg twice daily after 15 days in all patients. However, in 2 patients, after 2 and 4 weeks, respectively, of teriparatide 20 µg twice daily without calcium and calcitriol supplementation, the dose of the drug was reduced again to 20 µg once daily due to mild hypercalcemia, and 2 patients discontinued on visit 6 (week 9) and 7 (week 11) for adverse events, as described below.

Therefore, after 3 months of teriparatide treatment, 8 patients were on teriparatide 20 µg twice daily, and 2 patients 20 µg once daily (mean total teriparatide dose: 36 ± 8.4 µg daily).

At the end of 3 months of teriparatide treatment all 8 patients (100%), treated with 20 µg twice daily, reduced by at least 50% supplements with oral calcium carbonate and calcitriol. In 6 out of 8 patients (75%) calcium carbonate supplement was withdrawn, while calcitriol was suspended in 7 out of 8 patients (87%). Neither of the two patients assuming teriparatide 20 µg once daily eliminated or reduced at least 50% supplements of calcium and calcitriol to achieve normocalcemia. In the other 2 patients who discontinued treatment with teriparatide 20 µg twice daily after 4 and 6 weeks, calcitriol was totally withdrawn, and calcium carbonate was reduced by 50% in 1 patient and eliminated in the other patient.

The mean calcium carbonate dose before the start of therapy with teriparatide was 1450 ± 395.10 mg/day (range: 1000–2000) and decreased (in subjects who continued to take it) to 260 ± 362.70 mg/day (range: 0–1000) at the end of treatment with teriparatide, with a statistically significant difference compared to baseline. The mean dose of calcitriol before the start of therapy with teriparatide was 1.00 ± 0.26 µg/day (range: 0.50–1.25) and diminished to 0.12 ± 0.21 µg/day (range: 0–0.50) in subjects who continued to take it, with a statistically significant difference.

### Serum calcium levels

**Fig. 1:**
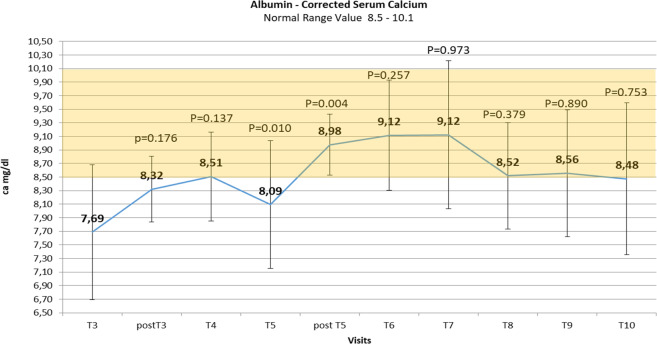
Changes over time in mean albumin corrected serum calcium levels ± SD (standard deviation) reported during the teriparatide treatment period. Number values are means, bars are SDs, and shaded area identifies the normal range of calcium level. Visit *T* (time) 3: first administration of teriparatide treatment 20 mcg/once daily; post T3: + 2–3 days; T4: +1 week; T5: +2 weeks, first administration of teriparatide 20 mcg/twice daily; post T5: +2–3 days; T6: +2 weeks; T7: +2 weeks; T8: +2 weeks; T9: +2 weeks; T10: +2 weeks

During the first 2 weeks of treatment with teriparatide 20 µg/once daily, 8 hypocalcemic episodes (calcemia < 8 mg/dL, mean value: 7.5 ± 0.2) without hospitalization in 6 patients out of 12 (50%) were described during the visits. Only after the beginning of teriparatide 20 µg twice daily, mean serum calcium concentrations tended to remain stable within the normal range (Fig. 1). However, during this period (weeks: 7–17), serum calcium oscillations were also reported. Six episodes of mild hypocalcemia (mean value: 7.40 ± 0.44 mg/dL) were described in 5 patients out of 10 (50%), and, in the same period, 7 episodes of mild hypercalcemia >10.1 mg/dL were described in 5 patients out of 10. For 2 of these patients, 1 after 2 weeks and another after 4 weeks of teriparatide 20 µg twice daily, the dosage was reduced definitively to 20 µg once daily for persistent mild hypercalcemia. The mean calcemia levels were higher (within normal range) at the end of teriparatide treatment (mean total teriparatide dose: 36 ± 8.4 µg/day) with a statistically significant difference as compared to baseline (Table 1).

### Serum phosphate and vitamin D levels

**Fig. 2:**
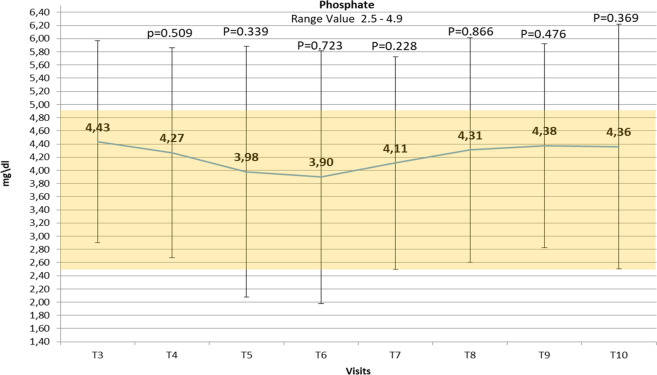
Changes over time in mean serum phosphate levels ± SD reported during the teriparatide treatment period. Number values are means, bars are SDs, and shaded area identifies the normal range of phosphate level. Visit *T* (time) 3: first administration of teriparatide treatment 20 mcg/once daily; post T3: +2–3 days; T4: +1 week; T5: +2 weeks, first administration of teriparatide 20 mcg/twice daily; post T5: +2–3 days; T6: +2 weeks; T7: +2 weeks; T8: + 2 weeks; T9: +2 weeks; T10: +2 weeks

The mean serum phosphate levels showed no statistically significant differences between the beginning and the end of teriparatide treatment (Table 1), remaining within the normal range both with 20 µg once daily and twice daily (Fig. 2). However, one episode of hyperphosphatemia during treatment with teriparatide 20 µg once daily in 1 patient, and 9 episodes of hyperphosphatemia (range: 5.1–6.3 mg/dL) in 3 patients during the treatment with teriparatide 20 µg twice daily, were registered. The mean serum calcium-phosphate product level was maintained within the normal range (<55 mg^2^/dL^2^) throughout teriparatide administration.

At the beginning of the teriparatide treatment, the mean 25(OH)D levels were above 20 ng/mL, but at the end of the treatment, a statistically significant decrease was shown. Mean 1,25(OH)_2_D_3_ level increased throughout the teriparatide treatment period, with a statistically significant difference at the end of treatment (Table 1).

The mean creatinine clearance level was >60 mL/min at the beginning of the teriparatide treatment period in all subjects. Throughout the study, a mild, but statistically significant, increase of creatinine clearance was observed between the beginning and the end of teriparatide treatment (Table 1).

### 24/h urinary calcium and phosphate

**Fig. 3:**
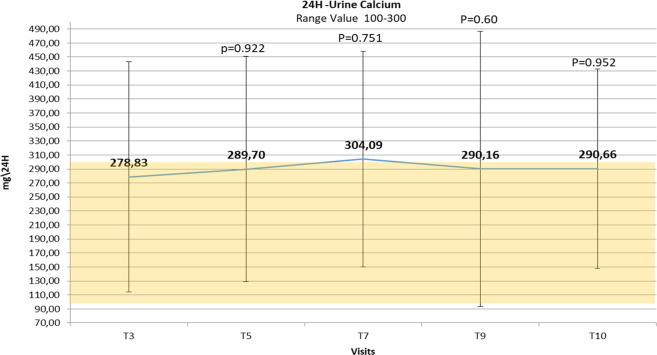
Changes over time in mean 24 h urine calcium levels ± SD reported during the teriparatide treatment period. Number values are means, bars are SDs, and shaded area identifies the normal range of urinary calcium level. Visit *T* (time) 3: first administration of teriparatide treatment 20 mcg/once daily; post T3: +2–3 days; T4: +1 week; T5: +2 weeks, first administration of teriparatide 20 mcg/twice daily; post T5: +2–3 days; T6: +2 weeks; T7: +2 weeks; T8: +2 weeks; T9: +2 weeks; visit T10: +2 weeks

The mean 24-h urinary calcium level (Fig. 3) was maintained within the normal range through the study, without statistically significant differences between the beginning and end of treatment with teriparatide. However, during the first 2 weeks of treatment with teriparatide 20 µg once daily, 6 episodes of hypercalciuria (>250 mg/24 h in females; >300 mg/24 h in the male) in 6/12 patients (50%) were described (not associated to hypercalcemia), and during the treatment with teriparatide 20 µg twice daily, 12 episodes of hypercalciuria not associated to hypercalcemia in 6/10 patients (60%) were demonstrated.

The mean 24-h urine phosphate level was within the normal range through teriparatide treatment (Table 1). Two episodes of hyperphosphaturia were reported with teriparatide 20 µg twice daily, not described with conventional treatment.

### Markers of bone remodeling

The mean bone alkaline phosphatase and urinary deoxypyridinoline levels, within the normal range at baseline, were significantly higher at the end of teriparatide treatment as compared to the beginning of treatment (Table 1).

### Quality of life evaluation

**Fig. 4:**
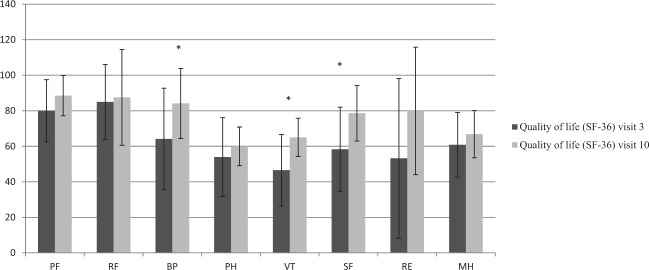
Changes over time in mean ± SEM (standard error of the mean) of SF-36 parameters evaluated during the teriparatide treatment period. Columns are means values of SF-36 score at visits 3 and 10; bars are for SEM. *P-value < 0.05. PF physical activity, RF role limitation caused by physical problems, BP bodily pain, PH perception of general health, VT vitality, SF social functioning, RE role limitations due to emotional, MH Mental health

The analysis of SF-36, performed at the beginning of teriparatide treatment and at the end, showed a statistically significant improvement of the quality of life in terms of “bodily pain” (BP), “social functioning” (SF), and “vitality” (VT), compared to the beginning of treatment, as shown in Fig. 4.

### Adverse events related to teriparatide and discontinuation

Two patients discontinued treatment with teriparatide 20 µg twice daily, one after 4 weeks and the other after 6 weeks. In both cases, teriparatide was discontinued for bone-joint pain in upper and lower limbs, plus headache in one case (mild grade), resolved upon discontinuation of the drug.

## Discussion

In some cases, the standard treatment of HypoPT can be challenging and can lead to long-term complications [1–3, 37–39]. The cause of this is the lack of a true replacement hormonal therapy, as multiple actions of PTH cannot be fully restored by calcium and calcitriol [2, 40–42].

Clinical studies with PTH (1–34) have been limited to small sample observations and prospective open-label studies [10–12, 14, 15, 20, 21], while an intervention randomized controlled phase III trial employing rhPTH (1–84), followed by further long-term prospective studies were conducted [32–35, 43–49].

The recommended daily doses for teriparatide and rhPTH (1–84) have mostly been based on the safety information collected in postmenopausal women, in whom the two drugs were approved for the treatment of severe osteoporosis at doses of 20 µg and 100 µg daily in adult patients, respectively [16, 22], and no safety issues related to osteosarcoma were raised [48, 50–52].

In our study, the patients selected on the basis of unsatisfactory response to conventional treatment were mainly affected by secondary HypoPT and most were females, in line with epidemiological data [38, 49, 53–56].

In this population, treated for 3 months, a dosage of teriparatide 20 µg once daily, in absence of calcium and calcitriol or with a reduction of at least 50% of calcium and calcitriol supplementation, did not allow the maintenance of normal serum calcium concentration. The dosage of 20 µg twice daily was followed by a better response, but it was also correlated to adverse events, such as high serum calcium levels, resulting in the need for a dose reduction to 20 µg once daily. Although the heterogeneity of the study designs with teriparatide does not make it possible to compare the results obtained on serum and urinary calcium levels [10–15, 20, 21, 57], some comments must be made.

The present data are supported by previous studies by KK Winer et al., showing that once-daily subcutaneous injection of PTH (1–34) (0.5 µg/kg·dose), could initially normalize mean serum and urine calcium levels in patients with HypoPT, but after 12 h, once-daily injection, even at dosages higher than 20 µg once daily, has been shown to have diminishing effects on serum calcium [12, 14]. A recently published investigation in 42 Italian adult hypoparathyroid patients showed that the use of teriparatide with a fixed-dose of 20 µg twice daily for 24 months was associated with mean stable calcium serum levels, although serum and urinary calcium fluctuations were also reported [21]. These results support previous observations by Winer et al. with PTH (1–34) administered twice daily at 46 or 37 µg/daily doses [10, 12, 15]. Altogether, these studies, in order to maintain stable mean calcium levels, doubled the recommended daily dose of PTH (1–34). Ideally, the last approach would be to use a pump delivery system by which PTH (1–34) [15], but intervention trials employing this method of administration are lacking.

The slight average increment in 24-h urinary calcium described in our study, and the episodes of hypercalciuria reported with teriparatide 20 µg twice daily through the 3 months of treatment, could be justified by the initial rise in bone turnover due to the action of PTH. This finding is in agreement with the results of previous studies [10, 21].

In this study, the mean serum phosphate levels remained within the normal range through the teriparatide treatment phase (with 20 µg either once or twice daily). However, some episodes of hyperphosphatemia were reported with teriparatide 20 µg twice daily. Similarly, episodes of hyperphosphatemia were also demonstrated in the prospective open-label 2-year study with teriparatide fixed-dose 20 µg twice daily [21], while no peaks in serum phosphate were demonstrated when PTH (1–34) was delivered by pump [10, 14].

In our study, as expected, the mean concentrations of 1,25(OH)_2_D_3_ increased at 3 months of treatment with teriparatide, while concentrations of 25(OH)D decreased, presumably due to the conversion of 25(OH)D, by the enzyme 1α-hydroxylase, to 1,25(OH)_2_D_3_, promoted by PTH. Similar results were also described in other studies employing teriparatide [20].

Regarding bone turnover status, this study showed that the levels of markers of bone turnover, after 3 months of treatment with teriparatide, increased with a statistically significant difference compared to baseline. Low-normal bone turnover is a typical feature of HypoPT [1, 37], and the use of teriparatide or rhPTH (1–84) can determine, especially in the first period of administration, a significant increase in markers of bone turnover due to an increased remodeling rate [10, 15, 27, 58].

Regarding adverse events, our study described mild events with 20 µg twice daily, such as bone-joint pain in upper and lower limbs, and headache, as similarly reported in previous investigations with PTH doses (1–34) >20 µg daily [10]. Other mild adverse events described in the prospective open-label study with teriparatide (fixed-dose 20 µg twice daily), such as myalgia and gastrointestinal illness [21], were not apparent in the patients treated in the present study.

Regarding the effect on the quality of life, in our study, teriparatide treatment improved significantly only some scores (BP, SF, and VT) of SF-36 at the end of 3 months, compared to baseline. The reasons for the improvement of some aspects of quality of life could be attributed to better compliance with subcutaneous therapy rather than multiple-daily oral therapies, and/or to a potential effect of PTH itself. On the other hand, the study conducted for 2 years with teriparatide fixed-dose 20 µg twice daily described an initial improvement (at 6 months) of all eight scores, but PF (physical functioning), RE (role limitations due to emotional health problems), and PH (Perception of general health) showed a subsequent significant reduction at 24 months compared to 6 months [21]. Long-term studies in larger patient populations should continue in the future, to clarify the effect of PTH peptides on quality of life, also considering the potential effects of PTH on the brain [41, 59, 60].

In conclusion, our investigation highlights the challenges related to the use of teriparatide in HypoPT at a dosage of 20 µg once daily, as currently recommended based on safety data. At this dosage and posology, teriparatide does not make it possible to avoid supplementation with calcium and calcitriol. On the other hand, teriparatide 20 µg twice daily can be associated with episodes of calcium and phosphate oscillations, and/or adverse events, although, since the small group of evaluated patients, this study does not allow conclusive results. Three months of treatment is not a long evaluation period, but it was sufficient to show that teriparatide with a fixed-dose of 20 µg/day was not able to maintain normocalcaemia by reducing calcium/calcitriol supplementation. A control group was not added as the goal of this study was to evaluate the efficacy and safety of both drug doses in the same patients. At last, regarding teriparatide, randomized clinical trials for HypoPT are lacking, and currently, the use of this drug in HypoPT patients should be limited to 20 µg daily, as no safety data are available for higher dosages even in the elderly population treated for 2 years. The present study conveys an important cautionary message to the endocrinologists for the current use of PTH peptides in HypoPT with dosages above those for which safety data have been collected.

## Authorship criteria

This work submitted for publication is original and has not been published other than as an abstract or preprint in any language or format and has not been submitted elsewhere for print or electronic publication consideration. All authors participated in the work in a substantive manner, in accordance with ICMJE authorship guidelines, and are prepared to take public responsibility for it. All authors consent to the investigation of any improprieties that may be alleged regarding the Work. Each author further releases and holds harmless the Endocrine Society from any claim or liability that may arise therefrom.

## Data Availability

All data have been analyzed and are available. All data analyzed during this study are included in this published article.
